# Visceral Arterial Aneurysms Complicating Endoscopic Retrograde Cholangiopancreatography

**DOI:** 10.1155/2013/515201

**Published:** 2013-09-25

**Authors:** Vinaya Gaduputi, Hassan Tariq, Anil Dev

**Affiliations:** Department of Medicine, Bronx Lebanon Hospital Center, 1650 Selwyn Avenue, Suit No. 10C, Bronx, NY 10457, USA

## Abstract

We report this case of a 74-year-old man with altered anatomy secondary to Billroth-II surgery who underwent endoscopic retrograde cholangiopancreatography (ERCP) for choledocholithiasis and subsequently developed severe diffuse abdominal pain with drop in hemoglobin. Patient was found to have hemorrhagic shock requiring aggressive resuscitative measures. Patient was found to have large peripancreatic hematoma secondary to bleeding from gastroduodenal and superior pancreaticoduodenal artery pseudoaneurysms. Gastroduodenal artery aneurysm is the rarest of all the splanchnic artery aneurysms, and to our knowledge this is the only reported case of a gastroduodenal artery pseudoaneurysm complicating ERCP.

## 1. Introduction

Visceral artery aneurysms (VAAs) are a variegated group of conditions with a wide range of manifestations and prognostic significance. While true aneurysms result from intrinsic vessel wall defects, pseudoaneurysms are often precipitated by underlying traumatic or inflammatory etiologies such as pancreatitis, vascular or laparoscopic interventions, and rarely even liver transplantation [1, 2]. Pancreaticoduodenal and gastroduodenal artery aneurysms together account for only 3.5% of all VAA [3]. Pancreatitis is reported to be the inciting agent in almost half of the cases [3, 4]. However, post-ERCP pancreatitis has not been implicated before, in the formation of these aneurysms.

## 2. Case Report

**Figure 1:**
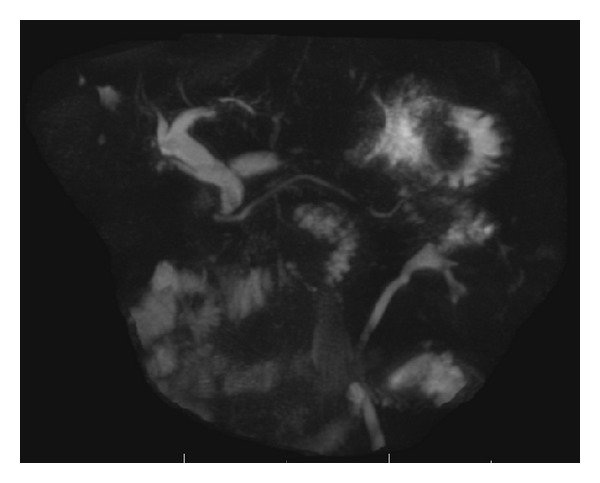
MRCP revealed dilated CBD with a large (1.5 cm × 0.55 cm × 0.5 cm) stone in the distal part.

A 74-year-old Hispanic man, who was born and raised in Dominican Republic, first presented to the gastroenterology clinic with complaints of epigastric and right upper quadrant abdominal pain. Physical examination at the time was unremarkable. Laboratory studies showed abnormal liver enzymes (alanine transaminase of 108 IU/L, aspartate transaminase of 87 IU/L, and normal alkaline phosphatase of 118 IU/L with normal bilirubin levels). Review of prior abdominal imaging from an outside facility revealed dilated common bile duct (CBD) of 1.5 cm. Patient was known to have Hepatitis C without cirrhosis and had failed treatment for it before. Patient had prior cholecystectomy for symptomatic cholelithiasis and Billroth-II surgery for complicated gastric ulcers. A magnetic resonance cholangiopancreatography (MRCP) done for the evaluation of dilated CBD revealed a large (1.5 cm × 0.55 cm × 0.5 cm) stone in the distal CBD (Figure 1). Patient was scheduled for an elective ERCP for stone removal.

During ERCP, the pancreatic duct was cannulated once, but CBD could not be cannulated in view of altered anatomy from Billroth-II procedure. ERCP was aborted. The procedure was planned to be reattempted and patient was discharged home on the same day.

Patient presented to the emergency room within a few hours with severe epigastric and right upper quadrant abdominal pain associated with nausea. Physical examination revealed a man in distress with stable vital signs and epigastric, right upper quadrant tenderness. Initial set of laboratory studies showed hemoglobin of 16.8 g/dL, abnormal liver enzymes (alanine transaminase of 122 IU/L, aspartate transaminase of 126 IU/L, normal alkaline phosphatase of 87 IU/L with elevated total and direct bilirubin levels of 1.8 mg/dL and 0.5 mg/dL resp.), normal renal function (BUN of 13 mg/dL and serum creatinine of 0.6 mg/dL), elevated amylase level of 1607 U/L, and elevated lipase level of 1275 U/L. A computerized tomogram of the abdomen revealed extensive peripancreatic inflammatory changes. Patient was admitted to the hospital with impression of post-ERCP versus biliary pancreatitis and was started on aggressive intravenous hydration.

**Figure 2:**
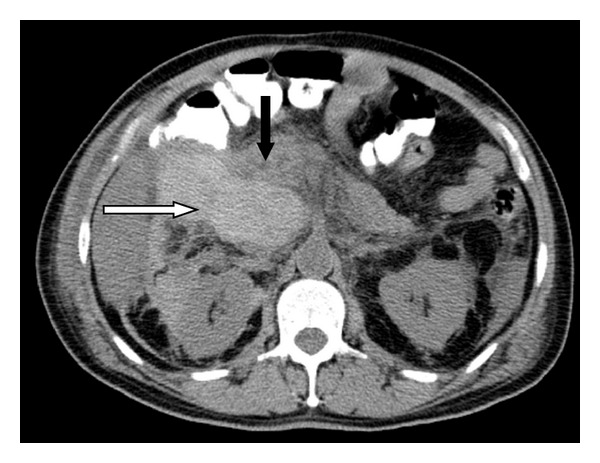
CT of abdomen revealed a large hematoma inferior and medial to the liver (white arrow) and abutting the pancreatic head (black arrow).

Over the next two days, patient complained of progressively worsening abdominal pain. Patient was found to have tachypnea, tachycardia with hypotension on day 3 of hospitalization. Abdominal examination revealed new-onset fullness and severe tenderness in the right upper quadrant region. Patient was found to have a precipitous drop in hemoglobin level to 7.1 gm/dL. The patient was transferred to critical care unit for management of hemorrhagic shock. CT of abdomen revealed a large hematoma (18 cm × 10 cm × 8.6 cm) inferior and medial to the liver extending through the foramina Winslow and abutting the pancreatic head (Figure 2). 

**Figure 3:**
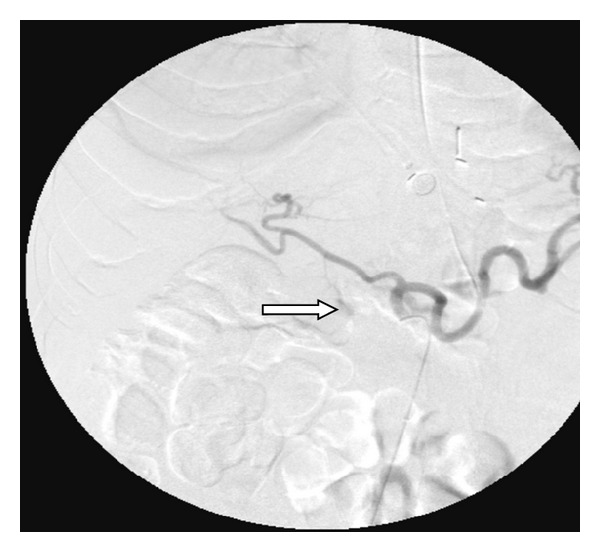
X-ray angiogram of the abdomen revealed pseudoaneurysms in the distal gastroduodenal artery.

An emergent CT angiogram of the abdomen revealed pseudoaneurysms in the distal gastroduodenal artery (Figure 3) and superior pancreaticoduodenal artery. Transcatheter embolization of the gastroduodenal artery was performed using Ivalon particles and 2-3 mm diameter platinum coil springs. The patient subsequently made full recovery and was discharged home. 

## 3. Discussion

ERCP is fraught with risk of multiple complications that could be broadly divided into general complications common to all endoscopic procedures (bleeding, perforation, untoward cardiopulmonary events, and risks of sedation) and specific complications that arise from pancreaticobiliary manipulations (pancreatitis, hemorrhage, and retroperitoneal duodenal perforation). Overall incidence of post-ERCP pancreatitis ranges from 1% to 6% in general population with rates approaching almost 30% in certain subsets of high-risk population [5]. Several mechanisms of development of post-ERCP pancreatitis have been proposed including mechanical trauma to the pancreatic duct [6] and hydrostatic and chemical damage from injected contrast [7, 8]. VAA formation is considered a very rare complication of ERCP, directly resulting from traumatic injury to the visceral arteries during pancreaticobiliary manipulations or indirectly from pancreatitis. Very few cases reports of ERCP directly leading to formation of VAA have been reported [9, 10]. However, all these cases involved maneuvers like sphincterotomy or stent placement being performed. We did not perform any aforementioned therapeutic maneuvers in our patient due to uncongenial anatomy as a result of prior Billroth-II surgery. We, therefore, attribute VAA formation in our patient to the underlying iatrogenic pancreatitis. 

Pseudoaneurysms result from injury to one or multiple layers of vascular wall from an underlying etiology such as acute pancreatitis. Acute pancreatitis could lead to pseudoaneurysm formation either by direct enzymatic destruction of vascular wall components or erosion from pseudocysts [11]. While overt gastrointestinal bleeding is the most common symptom of presentation of a gastroduodenal artery aneurysm, abdominal pain could be found in up to half of the patients [1]. Mortality estimates from a ruptured gastroduodenal artery aneurysm vary greatly depending on the size of aneurysm and site of rupture. An intraperitoneal bleed from gastroduodenal artery aneurysm as seen in our patient has a mortality rate of almost 19% [12, 13]. Gold-standard for the diagnosis of VAA and their rupture is visceral angiography [14].

To our knowledge, this case is amongst only few that represent VAA complicating ERCP and the only one that occurred without a therapeutic intervention being performed. The potential for rapid growth and increased chance of rupture with very high mortality rates make VAA a diagnosis that must be actively sought and aggressively managed in right clinical context such as that seen in our patient.
